# Fractional Exhaled Nitric Oxide Testing: Diagnostic Utility in Asthma, Chronic Obstructive Pulmonary Disease, or Asthma-chronic Obstructive Pulmonary Disease Overlap Syndrome

**DOI:** 10.7759/cureus.4864

**Published:** 2019-06-10

**Authors:** Jeffrey A Miskoff, Asa Dewan, Moiuz Chaudhri

**Affiliations:** 1 Internal Medicine, Jersey Shore University Medical Center, Neptune City, USA; 2 Miscellaneous, Hackensack Meridian Health, Neptune City, USA

**Keywords:** copd, acos, asthma, pulmonary, feno, obstructive, airflow limitation

## Abstract

Asthma and chronic obstructive pulmonary disease (COPD) can present as unique conditions or as a combination known as asthma-chronic obstructive pulmonary disease overlap syndrome (ACOS). These condition(s) can be categorized as obstructive conditions, causing inflammation of small airways leading to decrease airflow, mucus production, and bronchoconstriction. Asthma and COPD affect every age, gender, ethnicity, and socioeconomic status, thus increasing mortality and morbidity burden in our society. Fractional exhaled nitric oxide (FeNO) is an endogenous gaseous molecule which can be measured in the human breath test because of airway inflammation. It has been studied extensively as a marker of inflammation and has been incorporated into an algorithm for asthma management. The purpose of this study was to investigate whether FeNO testing can lead to a change in the diagnosis. A retrospective chart review of 95 patients with asthma, COPD, and ACOS was performed, and FeNO levels were recorded. Out of 95 patients, 36%, 24%, and 22% of the patients had an initial diagnosis of asthma, COPD, and ACOS, respectively. After the FeNO testing, the number of patients with the final diagnosis of asthma and ACOS increased, and COPD decreased. Our results support the utility of FeNO as a viable marker in diagnosing and managing complex cases of asthma, COPD, and ACOS.

## Introduction

Asthma and chronic obstructive pulmonary disease (COPD) are respiratory conditions which commonly present with airflow limitation; the patient often complains of experiencing shortness of breath. Asthma and COPD exacerbation present with increased airway inflammation, mucus production, and air trapping. Air trapping results from not being to exhale completely, leading to residual oxygen in the lungs. Data suggests that COPD is the third- and fourth-most cause of deaths in the United States and the world, respectively [1-3]. Some reports suggest that COPD will be the third leading cause of deaths in the world by 2020 [4-5]. COPD is a chronic, indolent condition with a prevalence of 10% in the general population and 50% in smokers. The average cost per simple and complex COPD exacerbation admission is 7242 dollars and 20757 dollars respectively. According to the Annals of the American Thoracic Society, asthma costs the United States 80 billion dollars every year [6]. High prevalence of these chronic ailments places a tremendous burden on our economy in the way of frequent office visits and hospitalization stemming from an exacerbation and thus requiring therapy such as supplemental oxygen and antibiotics [7].

Fractional exhaled nitric oxide (FeNO) is a gaseous molecule produced in response to an inflammatory process and may aid in differentiating asthma from other lung conditions [8]. This particle can be measured in a breath test, guiding the clinician in providing better care. Nitric oxide (NO) plays an important role in the immune system by controlling the vascular and bronchial tone [9]. In addition to asthma, FeNO has value in differentiating types of asthma from COPD and other conditions presenting with similar symptoms such as gastroesophageal reflux disease (GERD), vocal cord dysfunction, and eosinophilic bronchitis [10-11]. FeNO levels of < 25 of parts per billion (ppb) is considered normal, 25 ppb - 50 ppb as intermediate, and > 50 ppb high [9]. Although definitive data linking asthma and FeNO is lacking, some strong conclusions can be made, thus improving the correlation of FeNO to the condition in asthmatics. Research suggests that the patients with asthma generally have (1) higher concentration of NO detected in their breath test, (2) inflammatory insult leads to elevation of FeNO, (3) FeNO levels fluctuate due to hyper-inflammation leading to high FeNO levels [12-15]. The purpose of this study was to investigate the role of fractional exhaled nitric oxide (FeNO) testing and how it may aid in greater accuracy in the diagnosis of asthma, COPD, and asthma-chronic obstructive pulmonary disease overlap syndrome (ACOS).

## Materials and methods

**Figure 1:**
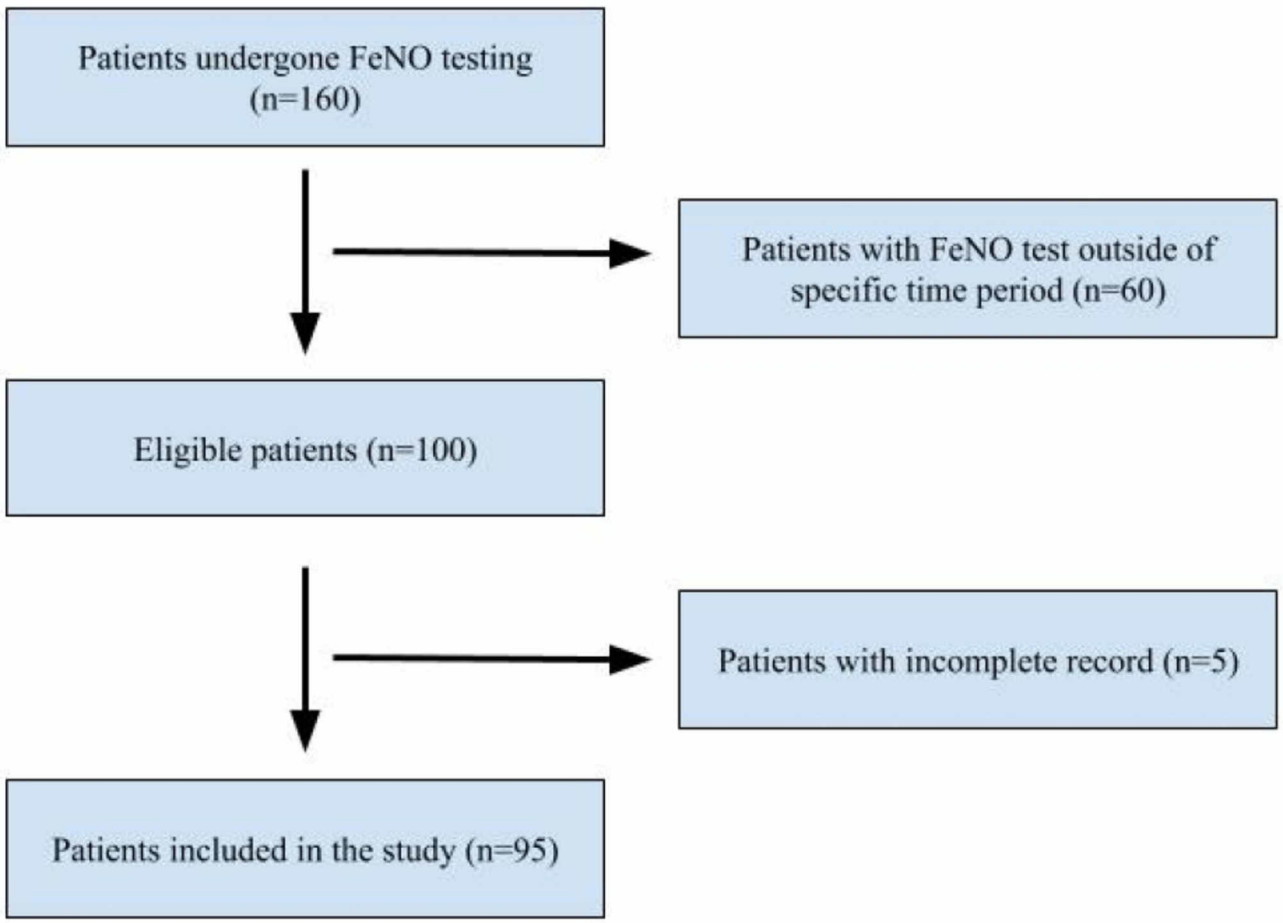
Study flow chart of patients in this study FeNO: fractional exhaled nitric oxide

We obtained records of all FeNO tests (160 patients) and results performed in the outpatient setting at our private practice affiliated with Jersey Shore University Medical Center (JSUMC). We focused on patients with the diagnosis of asthma and COPD who underwent FeNO testing between November 1, 2016, and February 28, 2017 (Figure 1). We identified 95 patients who satisfied the inclusion criteria; therefore, their electronic medical records (EMR) were reviewed. Date of the FeNO test was used to separate pre- and post-diagnosis. For example, if the patient had their FeNO test on November 15, 2016, then the diagnosis listed in their progress note before the FeNO test was documented as pre-FeNO diagnosis and diagnosis documented after the FeNO test was marked as a post-FeNO diagnosis.

Subsequently, their age, race, pulmonary function test (PFT) FeNO levels, diagnosis before and after the test, presenting symptoms, smoking history, and past medical history were recorded into an electronic spreadsheet (Microsoft Excel, Microsoft Office, Redmond, Washington, United States). Next, one of the authors calculated the mean and range of FeNO (ppb) levels along with identifying the change of diagnosis after the FeNO test.

## Results

Our analysis focused on patients who underwent FeNO testing starting November 1, 2016, to February 28th, 2017. We categorized diagnosis into asthma, chronic obstructive pulmonary disease (COPD), and asthma-COPD overlap syndrome (ACOS). For this study, diagnosis of COPD included patients who had chronic bronchitis and emphysema; bronchiectasis was not included in the diagnosis of COPD.

A total of 95 patients were included in this study (Table 1). Before FeNO testing, our cohort was characterized as 34 asthmatics, 23 with COPD, 21 with ACOS, and 17 patients without the diagnosis of asthma, COPD, or ACOS. The mean FeNO (ppb) level in patients with an initial diagnosis of asthma, COPD, ACOS, and without a diagnosis was 40.5 (ppb), 19.48 (ppb), 28.19 (ppb), and 12.38 (ppb), respectively.

**Table 1:** FeNO levels (mean) with the initial diagnosis FeNO: fractional exhaled nitric oxide; COPD: chronic obstructive pulmonary disease; ACOS: asthma-chronic obstructive pulmonary disease overlap syndrome; ppb: parts per billion

Condition	Mean FeNO (ppb) in initial diagnosis	FeNO (ppb) range in initial diagnosis
Asthma (n=34)	40.50	5 - 204
COPD (n=23)	19.48	5 - 46
ACOS (n=21)	28.19	6 - 96
No asthma, COPD or ACOS (n=17)	12.38	5 - 30
Total number of patients (n=95)		

After FeNO testing, our cohort was characterized as (Table 2): 45 asthmatics, 12 with COPD, 27 with ACOS, and 11 were undiagnosed. The mean FeNO (ppb) value in patients with the final diagnosis of asthma, COPD, ACOS, and without a diagnosis was 35.02 (ppb), 16.25 (ppb), 26.18 (ppb), and 10.27 (ppb) respectively. Furthermore, six patients, initially undiagnosed, were diagnosed with asthma and COPD as their final diagnosis based on their mean FeNO levels (Table 3). 

**Table 2:** FeNO levels (mean) with the final diagnosis FeNO: fractional exhaled nitric oxide; COPD: chronic obstructive pulmonary disease; ACOS: asthma-chronic obstructive pulmonary disease overlap syndrome; ppb: parts per billion

Condition	Mean FeNO (ppb) in final diagnosis	FeNO (ppb) range in final diagnosis
Asthma (n=45)	35.02	5 - 204
COPD (n=12)	16.25	5 - 33
ACOS (n=27)	26.18	6 - 96
No asthma, COPD or ACOS (n=11)	10.27	5 - 17
Total number of patients (n=95)		

**Table 3:** Pre-FeNO diagnosis vs post-FeNO diagnosis FeNO: fractional exhaled nitric oxide; COPD: chronic obstructive pulmonary disease; ACOS: asthma-chronic obstructive pulmonary disease overlap syndrome; ppb: parts per billion

Patient number	FeNO levels (ppb)	Initial diagnosis	Final diagnosis	Presenting symptom(s)
1	30	none	Asthma	Dyspnea
2	28	none	Asthma	Sarcoidosis, cough
3	8	none	Asthma	Dyspnea
4	25	none	Asthma	Dyspnea, cough
5	13	none	Asthma	Dyspnea, cough
6	26	none	ACOS	COPD exacerbation

In our cohort, one patient diagnosis changed from asthma to ACOS, nine patients with COPD were changed to the diagnosis of ACOS, two patients changed from COPD to asthma, and two patients were changed to having no final diagnosis despite having an initial diagnosis (Table 4).

**Table 4:** Changes from initial to final diagnosis FeNO: fractional exhaled nitric oxide; COPD: chronic obstructive pulmonary disease; ACOS: asthma-chronic obstructive pulmonary disease overlap syndrome; ppb: parts per billion

Patient number	Initial diagnosis	Final diagnosis	Presenting symptoms	FeNO (ppb) levels
1	Asthma	ACOS	Wheezing, cough	24
2	COPD	ACOS	n/a	27
3	COPD	ACOS	COPD exacerbation	6
4	COPD	ACOS	Dyspnea	19
5	COPD	ACOS	Cough	7
6	COPD	ACOS	n/a	18
7	COPD	ACOS	n/a	20
8	COPD	ACOS	Dyspnea	46
9	COPD	ACOS	COPD exacerbation	38
10	COPD	ACOS	n/a	21
11	COPD	Asthma	n/a	24
12	COPD	Asthma	n/a	28
13	COPD	n/a	Dyspnea, cough	12
14	COPD	n/a	n/a	10

## Discussion

Fractional exhaled nitric oxide (FeNO) is a marker of endogenous inflammation which can be used to monitor inflammatory changes in the airway. Asthma and COPD usually consist of airway inflammation and hyperresponsiveness. FeNO levels correlate with the presence of inflammation in the lungs; however, the precise role in diagnosis is not well defined. Despite lacking robust association, FeNO levels in conjunction with other laboratory markers and clinical signs aid in reaching a diagnosis [8].

In our cohort, 36% of the patients were initially diagnosed as asthmatics. However, this percentage increased to 47% after FeNO testing. Conversely, the number of patients initially diagnosed with COPD decreased following the FeNO testing (24% to 13%). Furthermore, the number of patients without an initial diagnosis decreased from 17 patients to 11 with five patients being diagnosed as asthmatics, and one patient as ACOS after FeNO testing (Table 3).

In a meta-analysis, 26 studies (4518) were reviewed, and the result suggests that FeNO is a reliable test to diagnose and manage asthma even though reported sensitivity and specificity was 0.65 and 0.82, respectively [16]. In a recent study, 197 patients with asthma, COPD, and ACOS were evaluated to differentiate ACOS from COPD using FeNO measurements. Their findings suggest that the patients with ACOS have higher mean FeNO levels than the patients with asthma (21.2 ppb vs. 13.0 ppb; P-value of 0.045) [17]. In a different case-control study, the authors focused on 53 patients diagnosed as ACOS and 53 patients who were categorized as non-ACOS. Results of the study show that the ACOS group had significantly higher FeNO levels than non-ACOS group (37 ppb vs. 20 ppb; P-value < 0.01) [18]. These results are consistent with our findings of observing high FeNO levels in the patients with ACOS as compared to non-ACOS (28.19 ppb vs. 19.48 ppb) group (Table 1). A similar trend of higher FeNO levels in ACOS group can be seen in our cohort presented in Table 2.

Although the clinical application of FeNO has increased over the years, it is important to evaluate all the available patient data before reaching a diagnosis. In Table 4, we presented patients with the initial diagnosis of asthma and COPD, which was changed without an elevated FeNO. Therefore, in addition to FeNO, other variables such as previous diagnosis, signs and symptoms, specific laboratory testing including immunoglobulin E (IgE), radioallergosorbent test (RAST), alpha-1 antitrypsin deficiency (AATD) genotype, spirometry, or response to therapies administered throughout their medical care was utilized to update their diagnosis.

Limitation of this study can be attributed to the FeNO test itself because of false positive results, although rare. This can occur with viral illness due to its ability to induce the production of nitric oxide in an attempt to utilize its antiviral properties [19]. Data suggest that specificity of this test is comparable to the bronchial challenge test, albeit being less sensitive [20]. Clinical evidence suggests that gender, age, and smoking influence FeNO levels. A study found that males with positive smoking history had lower FeNO levels than nonsmokers (P-value 0.001) [21]. Overall, we did not feel that our patient population was significantly affected by concurrent tobacco use or concomitant conditions.

## Conclusions

Fractional exhaled nitric oxide (FeNO) is a simple, reproducible, and a noninvasive marker of inflammation which may be used as one piece of data to help the clinician better assess the patients true underlying pulmonary disorder and potentially change the treatment plan. Although the primary purpose of this study was to help more accurately diagnose the patient, the true value of FeNO may be in regards to treatment. Therefore, further studies are needed to better understand FeNO and its applicability across the clinical spectrum.
